# P-445. Susceptibility and molecular characterization of isolates from a Phase 2/3, open-label, randomized, active-controlled clinical trial evaluating the safety and efficacy of imipenem/cilastatin/relebactam (IMI/REL) in pediatric participants with gram-negative (GN) bacterial infection

**DOI:** 10.1093/ofid/ofaf695.660

**Published:** 2026-01-11

**Authors:** Katherine Young, David W Hilbert, Carmelle Norice, Prachi Nair, Mark Estabrook, Christopher Bruno

**Affiliations:** Merck, Rahway, New Jersey; Merck Research Laboratories, Rahway, New Jersey; Merck & Co., Inc., North Wales, Pennsylvania; Merck & Co., Inc., North Wales, Pennsylvania; IHMA, Schaumburg, IL; Merck & Co., Inc., North Wales, Pennsylvania

## Abstract

**Background:**

Antimicrobial resistance among GN pathogens is a serious global public health issue, affecting adult and pediatric populations. New treatment options for carbapenem-resistant or ESBL-producing pathogens are needed.

**Figure:**
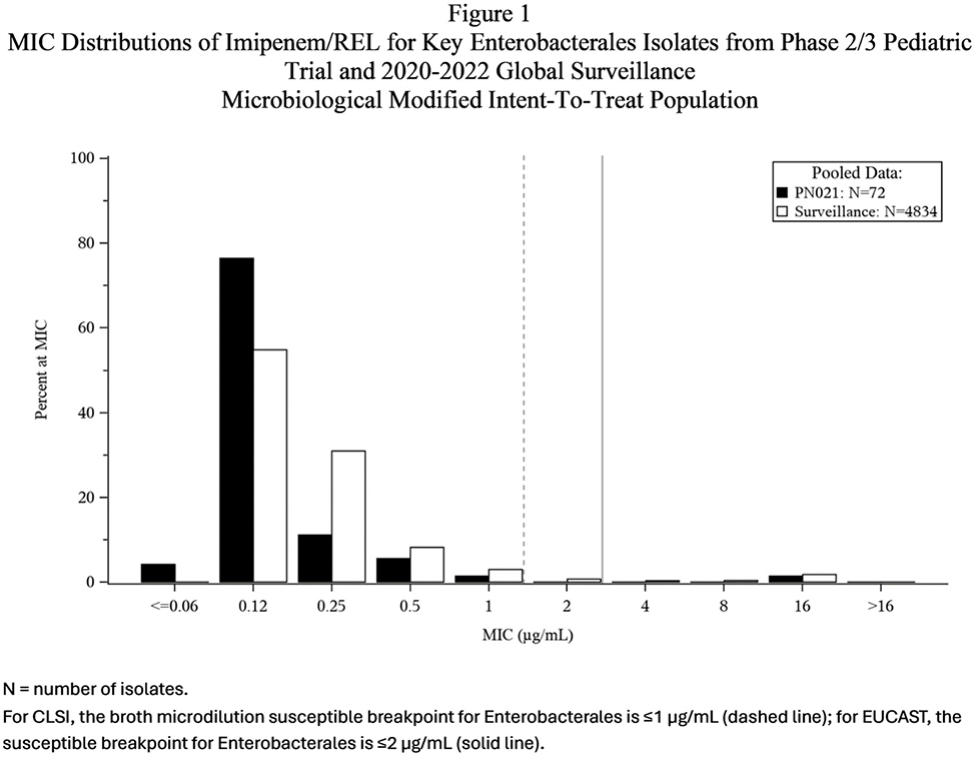

**Figure:**
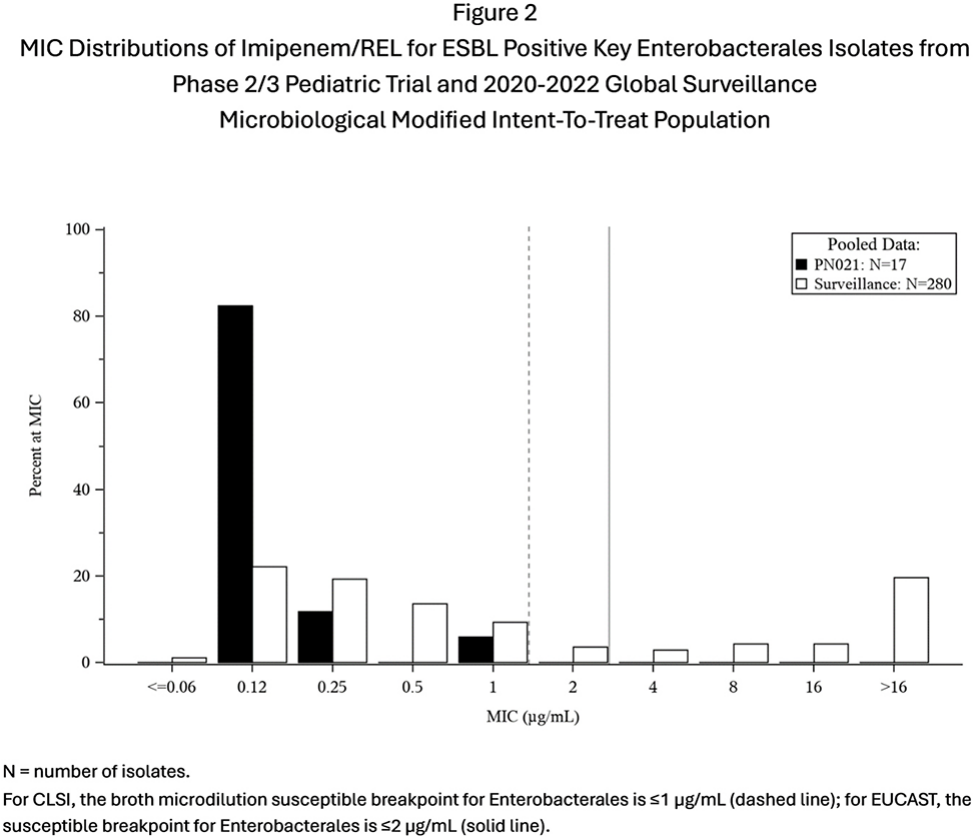

**Methods:**

This study was a Phase 2/3, open-label, randomized, active-controlled, multinational clinical study enrolling children (birth to < 18 years old) with confirmed or suspected hospital-acquired/ventilator-associated bacterial pneumonia, complicated intra-abdominal infection, or complicated urinary tract infection including pyelonephritis. Participants required hospitalization and intravenous antibacterial therapy and were randomized 3:1 to IMI/REL or active control. Bacterial pathogens isolated from primary infection sites were sent to a central laboratory (IHMA; Schaumburg, IL, USA) for testing by broth microdilution of IMI/REL using CLSI guidelines. Molecular mechanisms of resistance to IMI and/or IMI/REL were characterized; PFGE typing was used to determine relatedness of isolates. Susceptibility and molecular characterization of isolates were compared to surveillance data from SMART, a global surveillance study monitoring pathogen prevalence and resistance of GN bacteria.

**Figure:**
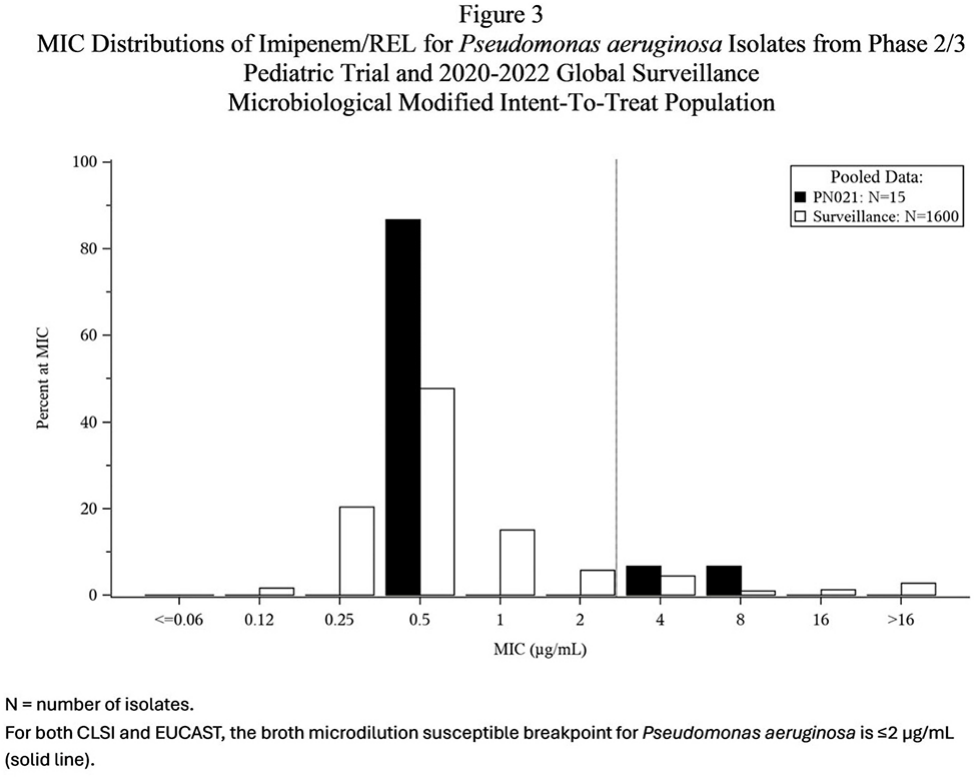

**Results:**

Of 115 participants randomized, 90 were in the microbiological modified intent-to-treat population (mMITT) population. MIC distributions from clinical trial and surveillance isolates were similar (Fig 1-4). IMI nonsusceptible (NS) baseline pathogens (7 Enterobacterales (non-Morganellaceae) (N=4) and *P. aerguinosa* (N=3)) from the mMITT population were evaluated for resistance determinants against β-lactams using PCR and sequencing. Of the 4 IMI NS Enterobacterales isolates evaluated, 3 were susceptible (S) to IMI/REL, (the isolates carrying KPC, AmpC, or no acquired β-lactamase were S), the isolate carrying NDM-1 was NS. Of the 3 IMI NS *Psa* isolates evaluated, 1 was S to IMI/REL, with 1 isolate carrying PDC-374 being S, and the isolate carrying the VEB-9 ESBL and 1 of the isolates carrying PDC-374 being NS.

**Conclusion:**

Isolates from this clinical trial were generally susceptible to IMI/REL with low MICs and representative of pediatric surveillance isolates.

**Disclosures:**

Katherine Young, M.S., Merck & Co., Inc.: Stocks/Bonds (Public Company) David W. Hilbert, PhD, Merck & Co., Inc.: Stocks/Bonds (Public Company) Carmelle Norice, MD, Merck & Co., Inc.: Stocks/Bonds (Public Company) Prachi Nair, BS, Merck & Co., Inc.: Stocks/Bonds (Private Company) Christopher Bruno, MD, Merck & Co., Inc.: Stocks/Bonds (Public Company)

